# Response to Letter to the Editor Regarding Sex-Specific Coronary Artery Calcium Score

**DOI:** 10.1016/j.jscai.2026.104277

**Published:** 2026-03-12

**Authors:** Denizhan Ozdemir

**Affiliations:** University of Rochester Medical Center/Strong Memorial Hospital, Rochester, New York

We appreciate Dr Budoff’s letter^1^ and his longstanding contributions to the coronary calcium literature. The studies he has cited are foundational, but they address different clinical questions and use different angiographic definitions than those used in our work.^2^ As a result, they are not directly comparable with the specific use case and end point we evaluated.

Our study addresses a contemporary, clinically practical problem: in patients undergoing coronary computed tomography angiography (CCTA), heavy calcification can produce blooming artifacts and lead to overestimation of stenosis severity. In that setting, clinicians need specificity–oriented coronary artery calcium (CAC) thresholds that may help rule in clinically significant obstructive disease and improve positive predictive value when CCTA is equivocal because of calcification. Accordingly, we compared CAC score (Agatston) with invasive coronary angiography using a definition of clinically significant disease that reflects current decision making: ≥50% stenosis in the left main or ≥70% stenosis in other major epicardial vessels. When available, intermediate or equivocal lesions were adjudicated with adjunctive intravascular imaging and/or physiologic assessment.

By contrast, much of the earlier angiography–correlated CAC literature cited in Dr Budoff’s letter was developed in symptomatic cohorts referred for invasive angiography and typically defined significant disease as ≥50% stenosis in any vessel—a definition that is deliberately sensitivity forward and well suited to ruling out disease but less aligned with a high–specificity rule-in objective or with left main–specific thresholds.

Several examples illustrate these differences:

1. Budoff et al^3^ evaluated symptomatic, angiography-referred patients and used ≥50% stenosis in any vessel as the angiographic definition.
2. Knez et al^4^ reported diagnostic performance across multiple absolute CAC thresholds and age-/sex-based percentile thresholds in symptomatic patients, demonstrating the expected sensitivity–specificity trade-off. The percentile framework is presented as age– and sex–stratified reference values rather than a single universal percentile cutoff, and the angiographic end point is not the same as major-vessel definition.
3. Budoff et al^5^ similarly used an angiography–referred symptomatic cohort and developed a continuous probabilistic model tied to a ≥50% stenosis definition.
4. Rumberger et al^6^ derived receiver-operating characteristic–based CAC cut points across stenosis severities in a smaller cohort (only 99 patients with ≥70% stenosis); while historically informative, it used end point definitions that differ from those applied in our study.

For these reasons, we believe our study should be interpreted as complementary to—rather than duplicative of—the prior literature. The novelty is not that CAC has been correlated with angiography, but that our work operationalizes a specificity–focused CAC thresholding approach using stenosis definitions commonly used to guide contemporary clinical decision making and to support interpretation when CCTA is limited by heavy calcification.

Finally, we would like to clarify that our study was not intended to be presented as the first or largest angiography–correlated CAC study, and we do not extrapolate these thresholds to asymptomatic screening populations. Our conclusions are confined to the symptomatic/high-risk clinical context in which CCTA and invasive angiography are being used for evaluation of suspected obstructive CAD.

## Declaration of competing interests

The author declared no potential conflicts of interest with respect to the research, authorship, and/or publication of this article.
